# Tumor-Induced Osteomalacia With Normal Fibroblast Growth Factor-23 (FGF23) and Idiopathic Hypercalciuria

**DOI:** 10.7759/cureus.20893

**Published:** 2022-01-03

**Authors:** Jassel A Velazquez-Navarro, Edgar Loya-Teruel, Mariana Rios-Gomez, Juan E Montes-Ramirez

**Affiliations:** 1 Department of Endocrinology, Hospital Angeles Chihuahua, Chihuahua, MEX; 2 Department of Nuclear Medicine, Hospital Angeles Chihuahua, Chihuahua, MEX; 3 Department of Internal Medicine, Hospital Regional de Pemex en Salamanca, Salamanca, MEX; 4 Department of Neurology, Hospital General de Mexico Dr. Eduardo Liceaga, Mexico City, MEX

**Keywords:** paraneoplastic syndrome, 18f-fdg pet/ct, fgf23, hypophosphatemia, tumor-induced osteomalacia

## Abstract

Tumor-induced osteomalacia (TIO) is a rare acquired paraneoplastic syndrome characterized by low serum phosphate, phosphaturia, inappropriately low/normal levels of serum calcitriol, and normal or elevated levels of fibroblast growth factor-23 (FGF23). Finding this mesenchymal tumor is challenging since it is usually benign, small, slow-growing, and is localized in the appendicular skeleton. We report a 58-year-old male patient who arrived at the endocrinology outpatient clinic due to slowly progressive low back pain and generalized weakness since the age of 48. On physical examination, only a reduced range of motion was noted. Laboratory tests revealed hypophosphatemia with normal parathyroid hormone (PTH) levels, normal serum calcium, high 24-hour urine calcium, normal 1,25-dihydroxyvitaminD levels, low renal threshold phosphate concentration (TmPO4/GFR), and high FGF23. The 18F-fluorodeoxyglucose positron emission tomography/computed tomography (18F-FDG PET/CT) reported a hypermetabolic extramedullary lesion in the C1-C2 vertebral bodies measuring 1.5 x 1.1 cm. Two months after the 18F-FDG PET/CT, complete excision of the cervical tumor was performed. The pathology ward reported a histiocytic mesenchymal neoplasm with accumulations of multinucleated giant cells compatible with phosphaturic mesenchymal tumor. After surgery, the patient’s hypophosphatemia was completely resolved. With this, the diagnosis of TIO was confirmed. The patient remains asymptomatic, with normal phosphate levels at one year of follow-up. Hypophosphatemia due to renal losses in an adult patient is a challenging diagnosis and one must consider TIO, autosomal dominant hypophosphatemic rickets, fibrous dysplasia, and even Fanconi syndrome. FGF23 can be extremely useful during the diagnostic approach since acquired dependent hypophosphatemia (FGF23 ≥ 30 RU/mL) highly suggests TIO. In this case report, we want to highlight the paramount importance of adequate tumor screening in adult patients with acquired FGF23-dependent hypophosphatemia. TIO is a reversible cause of hypophosphatemia with potentially disabling consequences if left untreated. These manifestations are non-specific (bone pain and muscle weakness), while others are progressive and severely disabling (bone deformities and multiple fractures). In this case report, we want to highlight the paramount importance of adequate tumor screening in adult patients with acquired hypophosphatemia, and the crucial lead that phosphate and vitamin D regulating hormones (FGF23) have for suspecting TIO.

## Introduction

Tumor-induced osteomalacia (TIO) is a rare acquired paraneoplastic syndrome characterized by low serum phosphate, phosphaturia, inappropriately low/normal levels of serum calcitriol, and normal or elevated levels of fibroblast growth factor-23 (FGF23). Finding this mesenchymal tumor is challenging since it is usually benign, small, slow-growing, and is localized in the appendicular skeleton. The cervical spine localization is extremely rare with only five cases reported to date [1]. We report a case of cervical spine TIO with idiopathic hypercalciuria.

## Case presentation

A 58-year-old male patient arrived at the endocrinology outpatient clinic due to slowly progressive low back pain and generalized weakness since the age of 48. The patient had a medical history of urolithiasis since the age of 18. There were no relevant hereditary traits, no bone fractures, no slow growth, or slow dentition. On physical examination, his blood pressure was 120/70 mmHg, height was 175 cm, weight was 80 kg, and only a reduced range of motion was noted.

Laboratory tests revealed hypophosphatemia (1.1 mg/dL; reference range: 2.4-4.5 mg/dL), with normal parathyroid hormone (PTH) level of 46 pg/mL (14-64 pg/mL), normal serum calcium level of 9.2 mg/dL (8.5-10.2 mg/dL), high 24-hour urine calcium level of 412 mg/dL (100-300 mg/dL), normal 1,25-dihydroxyvitamin D level of 18 pg/dL (5-91 pg/dL), low renal threshold phosphate concentration (TmPO4/GFR) level of 1.55 (2.2-3.4), and normal/high FGF23 level of 162 RU/mL (<180 RU/mL). General urine test excluded Fanconi syndrome. Thus, treatment with phosphate 250 mg per day, calcitriol 10 mcg per day, and cholecalciferol 5,000 U per day was started with a goal of 2.7-3.0 mg/dL of serum phosphate. The hypophosphatemia, normal/high FGF23, and low TmPO4/GFR strongly suggested renal losses, thus, a TIO had to be considered.

**Figure 1:**
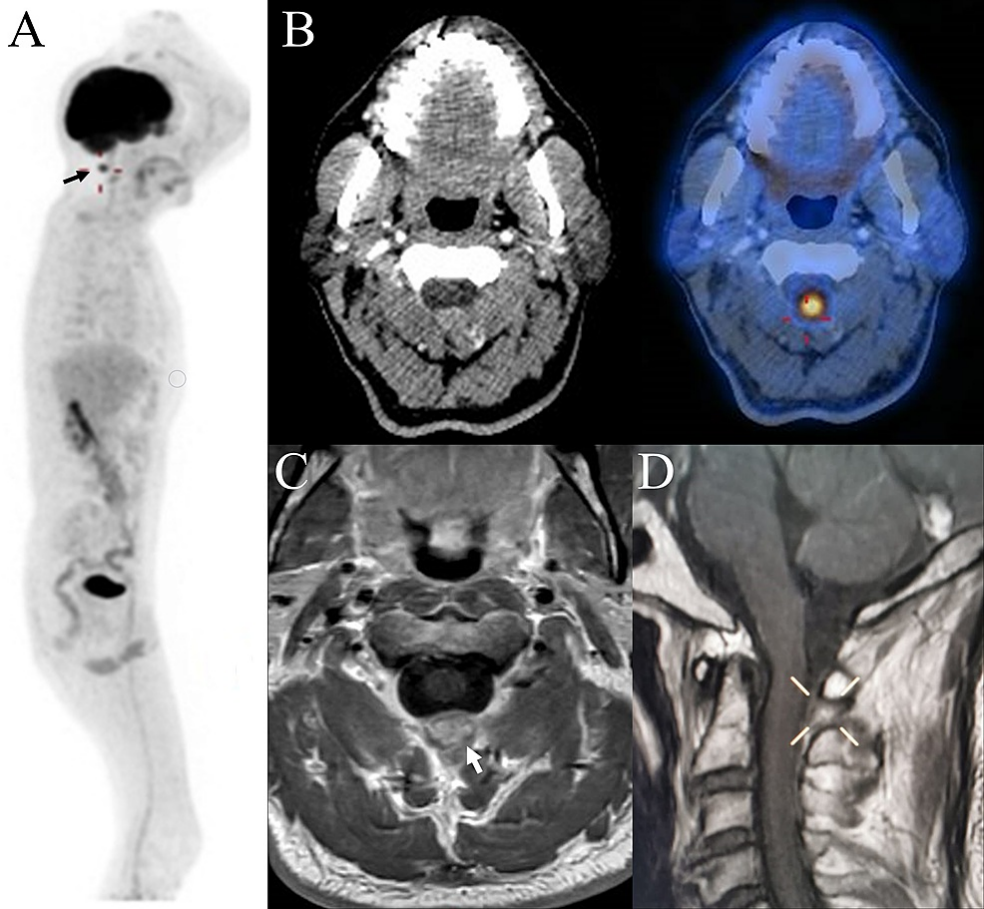
A: Planar lateral view of the 18F-FDG PET/CT showing a hypermetabolic lesion in C2 (black arrow). B: Axial CT scan and 18F-FDG PET/CT scan at C2 level showing the hypermetabolic lesion. C: Axial MRI scan at C2 level showing a hyperintense (1.6 x 0.7 cm) tumor (arrow). D: Sagittal MRI scan showing the hyperintense tumor. 18F-FDG PET/CT, 18F-fluorodeoxyglucose positron emission tomography/computed tomography.

The 18F-fluorodeoxyglucose positron emission tomography/computed tomography (18F-FDG PET/CT) reported a hypermetabolic extramedullary lesion (maximum standardized uptake value [SUVmax]: 12.4) in the C1-C2 vertebral bodies measuring 1.5 x 1.1 cm (Figures 1A, 1B). The magnetic resonance imaging (MRI) confirmed a C2 extradural, extramedullary (1.6 x 0.7 cm) hyperintense tumor (Figures 1C, 1D).

**Figure 2:**
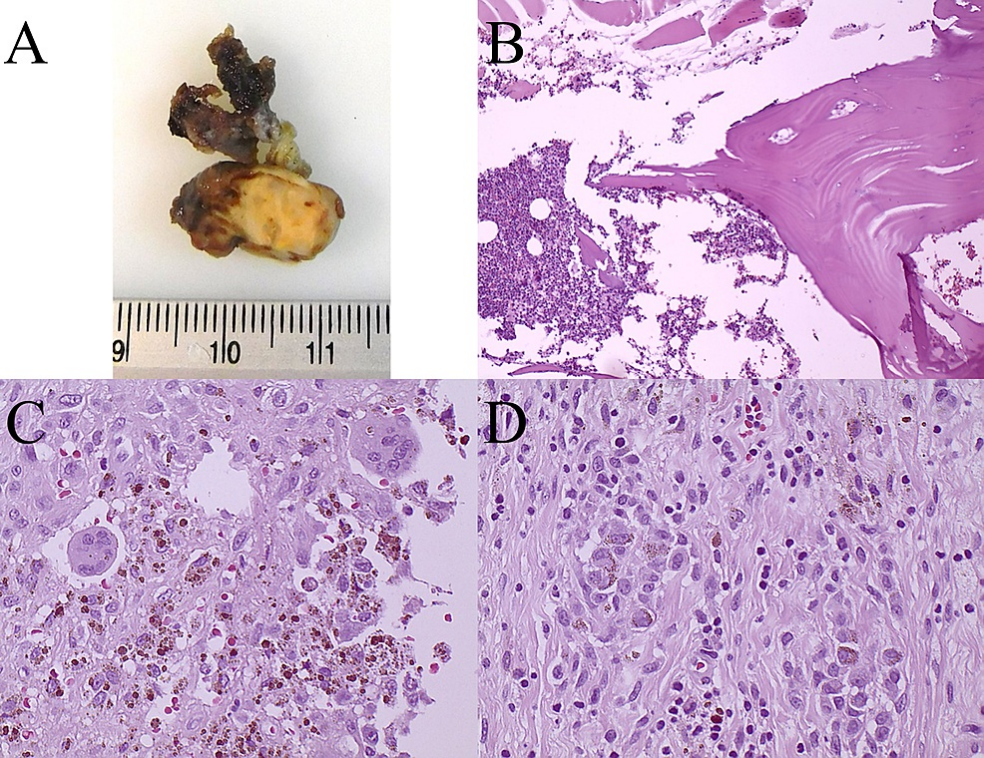
A: Macroscopic view of the 1.5 x 1.0 cm cervical tumor. B: Low power histological examination with hematoxylin & eosin staining. C: High-power view histological examination with hematoxylin & eosin staining. D: Histological examination with hematoxylin & eosin staining revealed a phosphaturic mesenchymal tumor with pigmented multinucleated giant cells.

Two months after the 18F-FDG PET/CT, complete excision of the 1.5 x 1.0 cm cervical tumor was performed (Figure 2A). The pathology ward reported a histiocytic mesenchymal neoplasm with accumulations of multinucleated giant cells compatible with phosphaturic mesenchymal tumor (Figures 2B-2D). After surgery, the patient’s hypophosphatemia was completely resolved. With this, the diagnosis of TIO was confirmed. The patient remains asymptomatic, with a normal phosphate level of 3.6 mg/dL (2.4-4.5 mg/dL) at one year of follow-up.

## Discussion

Phosphorus is an essential element for normal physiology, including intracellular signaling, membrane function, energy metabolism, and bone mineralization. Phosphate homeostasis is regulated by intestinal uptake, phosphate exchange between the extracellular and bone storage pools, and renal reabsorption [2,3].

Normally, 80% of the ingested phosphorus is absorbed by the intestine and its major regulator is renal reabsorption. The major regulators of renal reabsorption are PTH and FGF23 [4]. FGF23 is a 32 kDa glycoprotein mainly produced in bones by osteoblasts and osteocytes under physiological circumstances [5]. The principal actions of FGF23 on mineral metabolism are its suppressive effect on phosphate reabsorption from the urine by decreasing the expression of Na/Pi II and suppressing the synthesis of 1,25-dihydroxyvitamin D (1,25(OH)2D3) in the kidney [6].

Diseases with an excessive blood concentration of intact FGF23 lead to renal phosphate wasting and inappropriately low-circulating levels of 1,25(OH)2D3 in patients with normal kidney function. Human disorders associated with elevated intact FGF23 are autosomal dominant hypophosphatemic rickets (ADHR) with a defective cleavage site of FGF23, X-linked hypophosphatemic rickets (XLH), autosomal recessive hypophosphatemic rickets 1 (ARHR1) caused by overproduction of FGF23 in bone, and tumor-induced osteomalacia caused by FGF23-producing tumors [5].

Thus, FGF23 can be extremely useful during the diagnostic approach of a patient with hypophosphatemia. Independent hypophosphatemia (FGF23 levels < 30 RU/mL) is seen in cases of hereditary hypophosphatemic rickets with hypercalciuria (HHRH) and Fanconi syndrome. While dependent hypophosphatemia (FGF23 ≥ 30 RU/mL) is seen in TIO and ADHR, as in our patient [7].

The detection of a mesenchymal tumor with the resolution of the hypophosphatemia after resection confirms the diagnosis of TIO. Hence, the adequate screening of this small, slow-growing mesenchymal tumor is of paramount importance. We report the sixth case of cervical TIO to date [1,7-10].

## Conclusions

TIO is a reversible cause of hypophosphatemia with potentially disabling consequences if left untreated. These manifestations are non-specific, such as bone pain and muscle weakness. While other manifestations are progressive and severely disabling, such as bone deformities and multiple fractures. In this case report, we want to highlight the paramount importance of adequate tumor screening in adult patients with acquired hypophosphatemia, and the crucial lead that phosphate and vitamin D regulating hormones (FGF23) have for suspecting TIO.
